# Design of a Compact Space Search Coil Magnetometer

**DOI:** 10.3390/s26082415

**Published:** 2026-04-15

**Authors:** Yunho Jang, Ho Jin, Minjae Kim, Ik-Joon Chang, Ickhyun Song, Chae Kyung Sim

**Affiliations:** 1 School of Space Research, Kyung Hee University, Yongin 17104, Republic of Korea; toujour@khu.ac.kr (Y.J.); kimminjae11@khu.ac.kr (M.K.); 2 Korea Astronomy and Space Science Institute, Daejeon 34055, Republic of Korea; cksim@kasi.re.kr; 3 Department of Electronic Engineering, Kyung Hee University, Yongin 17104, Republic of Korea; ichang@khu.ac.kr; 4 Department of Electronic Engineering, Hanyang University, Seoul 04763, Republic of Korea; isong@hanyang.ac.kr; 5 Department of Astronomy and Space Science, University of Science and Technology, Daejeon 34113, Republic of Korea

**Keywords:** search coil magnetometer, space payload, magnetometer, rolling-sheet core, mass reduction

## Abstract

Search coil magnetometers (SCMs) are widely used in space science missions to measure time-varying magnetic fields. However, conventional SCM designs often increase sensor mass and electronic power consumption in order to meet mission-specific sensitivity requirements. This study presents the design and ground-based test results of a space search coil magnetometer (SSCM) concept aimed at reducing sensor mass and electronic power consumption while maintaining practical system operability for platform-constrained missions. Mass reduction was achieved by adopting a rolling-sheet core configuration. In addition, printed circuit board (PCB)-based interconnections between segmented windings were implemented to improve the reproducibility of assembly and mechanical robustness without additional structural complexity. Power reduction was achieved by employing an application-specific integrated circuit (ASIC)-based sensor amplifier and a compact control electronic unit implemented as a modular stack with a 1U CubeSat standard board form factor. Performance tests confirmed the stable operation of the integrated sensor–electronics chain over the target measurement band. The system-level noise-equivalent magnetic induction (NEMI) measured under laboratory conditions was 33 fT/√Hz at 1 kHz. Environmental tests including vibration and thermal cycling were performed to further verify the structural safety and functional stability of the sensor assembly under space-relevant conditions. The proposed SSCM architecture provides a practical approach for implementing low-mass and low-power magnetic field instruments for platform-constrained space missions.

## 1. Introduction

Magnetic field measurements are essential for interpreting electromagnetic phenomena in natural and engineered systems. The search coil magnetometer (SCM) senses time-varying magnetic fields and its high sensitivity and simple structure have supported its broad use in industrial and scientific fields, including medical instrumentation, electric machinery, and geophysical surveys [1,2,3].

In space science, SCMs are used to observe electromagnetic wave phenomena. Space plasma waves play a critical role in energy transfer and particle acceleration and significantly influence the formation and variability of radiation belts [4]. These waves are generated by plasma instabilities, which are driven by anisotropic velocity distributions in the presence of a background magnetic field, and they propagate either along the field lines or at oblique angles [5]. Electromagnetic waves—observed from the millihertz range of ultra-low-frequency (ULF) waves to the kilohertz range of whistler-mode chorus, magnetosonic waves, and plasmaspheric hiss—contribute to the acceleration, scattering, and atmospheric loss of high-energy radiation belt particles [6,7,8,9,10]. Therefore, space magnetic field observations provide essential information for understanding the dynamics of space plasma. Magnetometers have been consistently used in space science missions and will continue to serve as important instruments in the future. Among these instruments, SCMs have been widely used as essential tools for measuring time-varying magnetic fields in space environments and are expected to remain indispensable for future space missions. However, despite their importance, a systematic design methodology that simultaneously addresses mass reduction, power efficiency, and broadband operation is yet to be developed.

To understand the challenges of SCM design within space exploration missions, it is necessary to consider the complementary roles of SCMs and fluxgate magnetometers across different frequency regimes. In many space missions, magnetic field payloads employ fluxgate magnetometers and SCMs, which are complementary [11,12,13,14]. Fluxgate sensors accurately measure DC magnetic fields and low-frequency variations typically below several tens of hertz. However, due to limitations in the dynamic response of their magnetic core, their sensitivity decreases significantly at frequencies above several hundred hertz, with an effective sensitivity limit reported near 400 Hz [15,16]. These characteristics limit the capability of fluxgate sensors to observe high-frequency electromagnetic waves occurring in the range from several hundred hertz to several kilohertz, such as whistler-mode chorus, magnetosonic waves, and plasmaspheric hiss. In contrast, SCMs measure time-varying magnetic field components with high sensitivity and extend the observable frequency range up to several tens of kilohertz, thereby complementing the high-frequency limitations of fluxgate sensors [17,18]. However, the design of SCMs is fundamentally constrained by trade-offs between sensitivity, bandwidth, and sensor mass.

SCMs consist of a ferromagnetic core and a multi-turn winding; they measure AC magnetic field components through the voltage induced in the coil when the magnetic flux in the core varies with a time-varying magnetic field. Core properties, such as effective permeability and material, as well as the number of turns, are key parameters that determine SCM performance. Increasing effective permeability and the number of turns enhances the induced voltage and improves sensitivity. However, these design choices simultaneously increase sensor mass and reduce the effective frequency bandwidth, thereby introducing inherent trade-offs in SCM design.

Ground-based SCM systems operate with relatively fewer constraints on mass, volume, and power consumption. Our previously developed ground-based SCM aimed to observe ULF waves below several hertz in Antarctica. It used a large core and many windings to measure low-frequency signals with high sensitivity, resulting in a sensor mass exceeding 30 kg [19]. In contrast, spaceborne SCM systems must satisfy strict constraints on mass, volume, and power consumption while still meeting scientific observation requirements. These constraints are particularly critical for small satellite platforms such as CubeSats. As a result, conventional design approaches that prioritize sensitivity through increased core size and winding turns become less suitable for modern space missions. Consequently, spaceborne SCM designs have evolved toward satisfying scientific requirements while addressing platform constraints.

Against this backdrop, this study presents a design methodology for a compact space search coil magnetometer (SSCM) aimed at reducing sensor mass and power consumption under platform constraints. This work therefore focuses on establishing a system-level design methodology that integrates sensor structure, analog front end, and digital processing into a compact and resource-efficient architecture, rather than optimizing a single component. The system-level requirements of the SSCM are defined to observe electromagnetic wave phenomena from several tens of hertz to several kilohertz in the near-Earth and lunar environments. The proposed approach integrates a rolling-sheet core configuration to reduce sensor mass and application-specific integrated circuit (ASIC)-based electronics to achieve power-efficient signal amplification and processing. This design addresses the fundamental trade-offs among sensitivity, bandwidth, and sensor mass identified in conventional SCM systems. This paper presents the overall system architecture of the SSCM and evaluates its performance.

Although the SSCM is not intended as a flight model for a specific mission, the proposed design demonstrates a practical approach for reducing mass and power consumption in future spaceborne SCM systems. The remainder of this paper is structured as follows. Section 2 describes the SSCM requirements and system configuration. Section 3 presents the test methods used for system evaluation. Section 4 reports the experimental results. Finally, Section 5 summarizes the SSCM system and the main findings of this study.

## 2. Instrument Design

The scientific objective of the SSCM is to observe electromagnetic waves in diverse space regions, including the near-Earth region and in the vicinity of the Moon. In near-Earth space, plasma wave phenomena typically measure in the range of approximately 10 Hz to several kilohertz, with amplitudes of tens to hundreds of picotesla. Around the Moon, wave activity is mainly observed to occur in the region of tens to hundreds of hertz, with amplitudes reaching up to several nanotesla.

Based on these scientific objectives, the SSCM development requirements were defined by considering both observational needs and platform constraints. The system is designed to measure magnetic fields over a frequency range from 10 Hz to 20 kHz and to achieve a noise-equivalent magnetic induction (NEMI) of 1 pT/√Hz at 1 kHz.

The SSCM frequency range is comparable to the frequency ranges adopted in previous near-Earth space missions. This range covers key wave phenomena observed in near-Earth space, including magnetosonic, plasmaspheric hiss, and whistler-mode chorus waves. This requirement imposes design constraints on the sensor configuration, particularly in balancing sensitivity and bandwidth, which are inherently coupled in SCM systems.

In contrast, we defined the NEMI requirement by considering both the ground test conditions and the expected amplitudes in relation to our objectives. Laboratory environments, residual magnetic field variations, electromagnetic interference in test facilities, and uncertainties associated with magnetic shielding limit the verification of absolute noise levels at the fT/√Hz scale typically reported for spaceborne SCMs. Therefore, the NEMI requirement of the SSCM serves as a practical criterion for validating the operational stability of the compact design and the functionality of the signal processing chain, rather than as a flight-level sensitivity requirement. In addition, the NEMI value at 1 kHz is derived from the measured system output noise and transfer function under controlled laboratory conditions, enabling consistent relative performance evaluation under ground test limitations. The measurement was repeated multiple times under identical conditions, and the resulting spectra were averaged to improve statistical reliability and reduce the influence of transient environmental noise.

**Figure 1:**
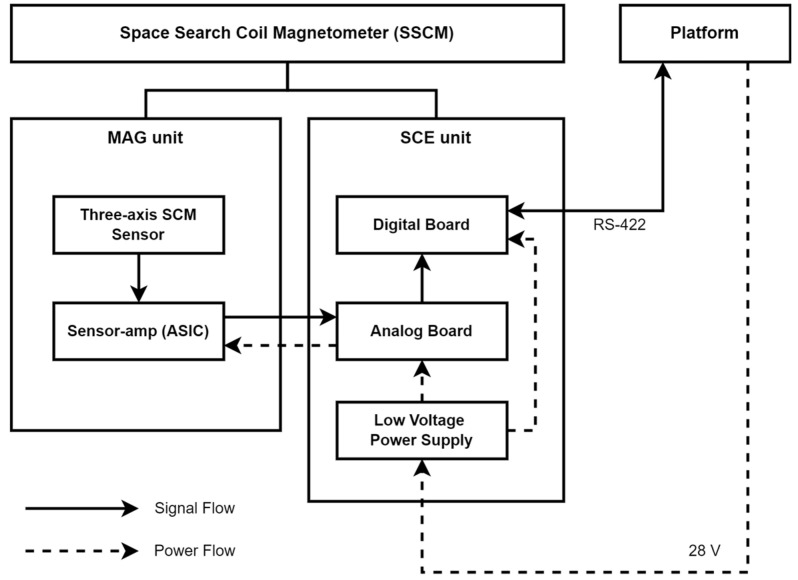
Block diagram of the space search coil magnetometer (SSCM).

Table 1 summarizes the system requirements of the SSCM. The SCM consists of a MAG unit and a search coil control electronics (SCE) unit—a block diagram of the SSCM system is shown in Figure 1.

### 2.1. MAG Unit

The MAG unit consists of a three-axis SSCM sensor and a sensor amplifier (sensor-amp). The unit is mounted on a boom to reduce bus-generated magnetic interference during wave measurements. This configuration is selected to ensure accurate wave measurements by minimizing spacecraft-induced magnetic disturbances, which is critical for high-sensitivity broadband observations.

**Figure 2:**
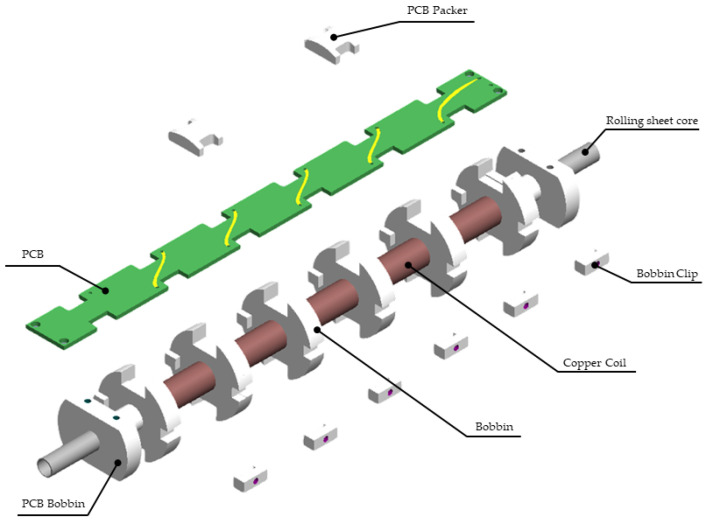
Exploded view of the SSCM sensor structure. Copper windings are wound around five bobbins that are secured using bobbin clips Each bobbin clip includes bolt holes that allow mechanical attachment to the CFRP case and the three-axis structure. Electrical connections between windings are implemented through a printed circuit board (PCB). The PCB is bolted to PCB bobbins located at both ends of the assembly, while two PCB packers positioned above the PCB restrict vertical motion. The magnetic core penetrates all bobbins and adopts a rolling-sheet core configuration.

The SSCM sensor employs a core length of 230 mm and a total of 12,000 turns. These parameters were determined based on the trade-off between performance and sensor mass, as increasing the number of turns and core size enhances the induced voltage while simultaneously increasing mass and limiting the effective frequency bandwidth. Figure 2 shows an exploded view of the SSCM sensor structure. Copper windings are wound around five bobbins that form the main sensing element of the search coil sensor. Each bobbin is mechanically fixed using a bobbin clip, and the clips include bolt holes that allow the entire assembly to be attached to the carbon fiber-reinforced plastic (CFRP) case and the three-axis structure. Electrical connections between the windings of adjacent bobbins are implemented using a printed circuit board (PCB). The PCB is mounted to PCB bobbins located at both ends of the structure and is constrained by two PCB packers positioned above the board to prevent vertical movement. This segmented bobbin configuration was adopted to reduce parasitic capacitance while maintaining structural stability, and the PCB-based interconnection improves assembly reproducibility. A rolling-sheet magnetic core penetrates all bobbins and forms the magnetic path of the sensor.

**Figure 3:**
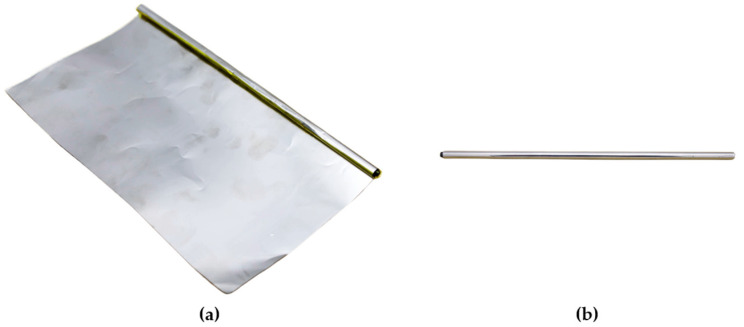
Rolling-sheet core configuration: (**a**) rolling-sheet core before winding; (**b**) rolling-sheet core.

Search coil cores are typically fabricated from ferromagnetic metals such as iron, nickel, or cobalt to obtain high magnetic permeability, which significantly contributes to sensor mass. In order to reduce the sensor mass, the SSCM adopts a rolling-sheet core configuration. The rolling-sheet core consists of annealed ferromagnetic sheets with a relative permeability of approximately 250,000, which are wound around a CFRP rod. Commercially available annealed ferromagnetic sheets were used. Figure 3 shows the rolling-sheet core. Compared with the conventional rod-type cores used in spaceborne SCMs, the rolling-sheet configuration reduces core mass from 60 g to 15 g while maintaining comparable sensitivity and measurement bandwidth [20]. Relative to a conventional rod-type core with a typical mass of approximately 60 g, the rolling-sheet core used in the SSCM has a mass of about 15 g, corresponding to a mass reduction of approximately 75% while preserving a similar measurement performance. This reduction directly mitigates the trade-off between sensitivity and sensor mass. In addition, the rolling-sheet core enables flexible tuning of sensor characteristics by modifying the sheet configuration and CFRP rod geometry, providing additional design adaptability. However, this core type is susceptible to fabrication-induced variations and its structural stability under space environmental conditions must be verified. To address this limitation, multiple rolling-sheet cores were fabricated and evaluated through frequency response tests, and three cores exhibiting consistent response characteristics were selected for the three-axis sensor configuration. Structural stability under space-relevant conditions was verified through thermal cycling and vibration tests. The detailed test procedures and results are presented in Section 3 and Section 4, respectively.

The sensor performance is influenced by the number of winding turns and the coil area. Increasing the number of turns enhances sensitivity but reduces the measurable frequency range and increases sensor mass. Therefore, the SSCM’s winding number was selected by balancing sensitivity, bandwidth, and mass constraints. Based on theoretical analysis and repeated frequency response tests with varying winding numbers, 12,000 turns were selected for the SSCM sensor.

**Figure 4:**
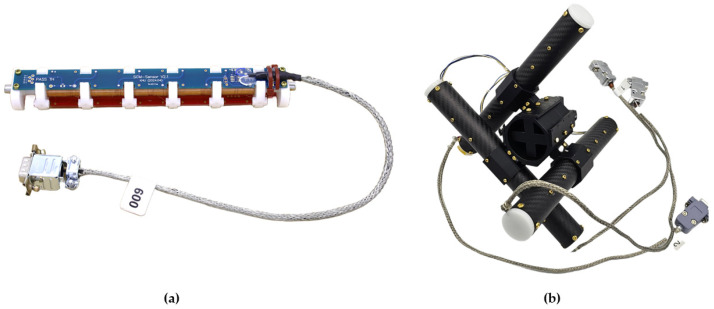
(**a**) SSCM sensor; (**b**) three-axis structure.

The total winding number was evenly distributed across five bobbins to reduce parasitic capacitance [17]. The bobbins were interconnected through a PCB that provides both electrical routing and mechanical stabilization of the winding assembly. The PCB also provided space for mounting temperature sensors; data from these sensors support on-orbit calibration. The PCB layout allows amplification circuitry to be integrated within the sensor assembly; this configuration conditions the signal before transmission to the electronics unit. Figure 4 shows the SSCM sensor and the three-axis structure.

The sensor-amp is installed on the boom structure near the SSCM sensor and is exposed to the space environment. The design maintains stable gain under radiation and large temperature variations. The sensor-amp boosts tens to hundreds of microvolts of signals generated by science-driven phenomena in space and forwards the signal to the SCE unit.

**Figure 5:**
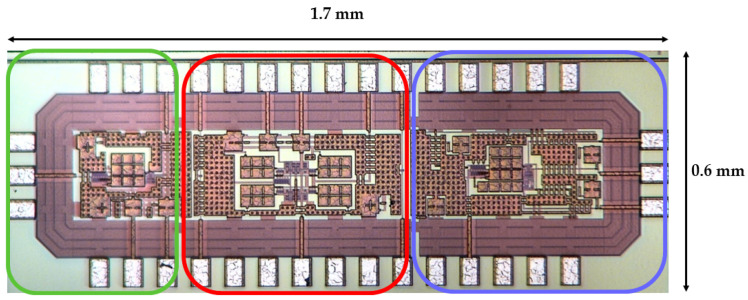
Sensor-amp ASIC chip (1.7 × 0.6 mm). The green region corresponds to the amplification circuit, the red region to the TID-hardening circuit, and the blue region to the SEE mitigation circuit.

The SSCM is integrated with an application-specific integrated circuit (ASIC)-based amplification circuit for low-power operation. The ASIC was fabricated using a 180 nm TSMC process, with a die size of 1.7 mm × 0.6 mm. The sensor-amp uses a two-stage architecture that balances voltage gain and bandwidth, where each stage provides 40 dB gain. The ASIC integrates radiation-tolerant circuit blocks and a temperature compensation feedback loop to mitigate gain drift over temperature [21]. Figure 5 shows the sensor-amp ASIC chip. The ASIC is divided into three functional regions, with the amplification chain implemented in the left region, and the middle and right regions providing total ionizing dose (TID) hardening and single-event effect (SEE) mitigation circuitry, respectively.

**Figure 6:**
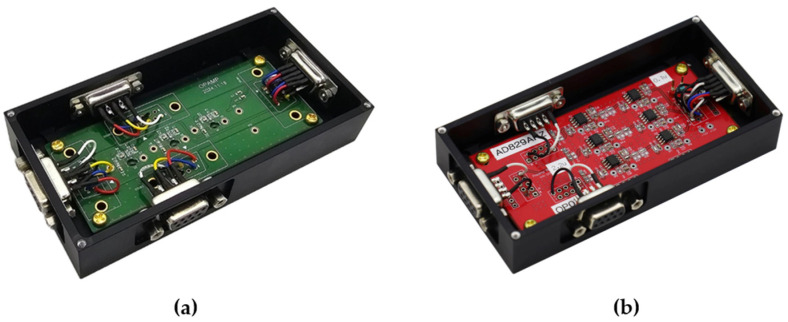
Sensor-amp and commercial OP-amp comparator: (**a**) ASIC sensor-amp; (**b**) comparator.

A reference amplifier based on commercial OP-amps was built as a comparator to evaluate the power consumption of the ASIC-based sensor-amp. Both amplifiers were designed to operate with the same voltage gain and bandwidth. The SSCM configuration was identical except for the sensor-amp. Power was measured with a 28 V input and the system consumed 3.92 W with the ASIC sensor-amp and 4.48 W with the comparator; this result corresponds to a 12.5% reduction in power consumption and supports the implementation of the SSCM in power-limited space platforms. Figure 6 shows the configurations of the sensor-amp and the comparator.

A parallel damping resistor was placed at the sensor input to prevent ADC saturation in the resonance band. This approach supports a stable frequency response over 10 Hz to 20 kHz. Shielded D-sub interfaces and harnesses were used to reduce external interference. The ASIC sensor-amp and the comparator used the same PCB footprint of 45 × 100 mm to ensure a fair power comparison. The ASIC design leaves additional board area in this prototype; however, a smaller PCB and housing may be achievable after layout and interface optimization, which would result in mass savings for space payload integration.

### 2.2. SCE Unit

**Figure 7:**
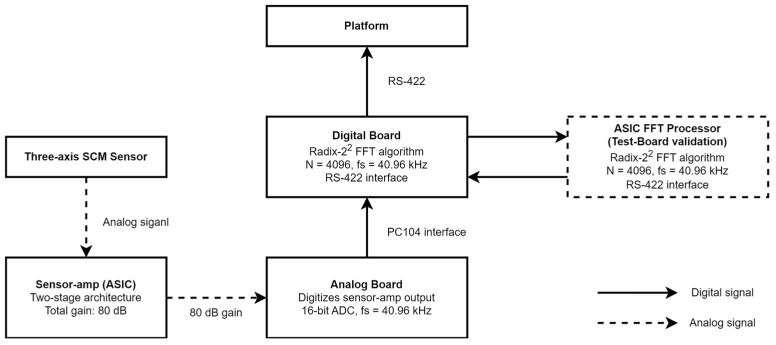
Data processing flow diagram.

The SCE unit is located inside the platform and controls the MAG unit and converts sensor signals into digital data for delivery to the platform. It adopts a three-layer stack consisting of a low-voltage power supply (LVPS), an analog board, and a digital board. Each board follows the 1U CubeSat standard board size and connects through a PC 104 interface. Figure 7 shows the data processing diagram.

The LVPS is placed on the bottom layer. It receives 28 V from the platform and converts the input to the voltages required by the SSCM, while operating over a 24 V to 32 V input range. The design follows the LVPS heritage of KMAG installed on the Korean lunar orbiter, Danuri.

The analog board forms the middle layer and digitizes the analog output from the sensor amplifier. The board integrates a 16-bit ADC with a sampling rate of 40.96 kHz. An offset circuit shifts the sensor amplifier output of ±2.5 V into the ADC input range of 0 V to 5 V.

**Figure 8:**
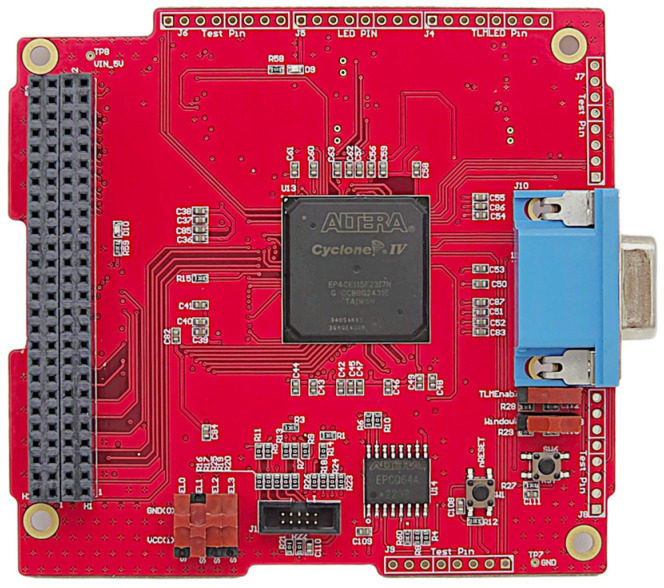
Digital board configuration (95.89 × 90.17 mm).

The digital board is placed on the top layer and uses an RS-422 interface for data transmission and command reception. Differential signaling improves the robustness against harness noise and supports reliable communication with the platform [22]. The digital board also performs fast Fourier transform (FFT) processing on the acquired signals using an ALTERA Cyclone IV Field Programmable Gate Array (FPGA). It applies a Radix-2^2^ algorithm to achieve faster processing than Radix-2 while reducing power consumption compared to Radix-4 [23]. The frequency resolution Δf depends on the sampling frequency fs and the FFT length N. The digital board sets fs to 40.96 kHz and N to 4096; this configuration provides a frequency resolution of approximately 10 Hz. A Hamming window suppresses spectral leakage during FFT processing. Figure 8 shows the digital board configuration.

**Figure 9:**
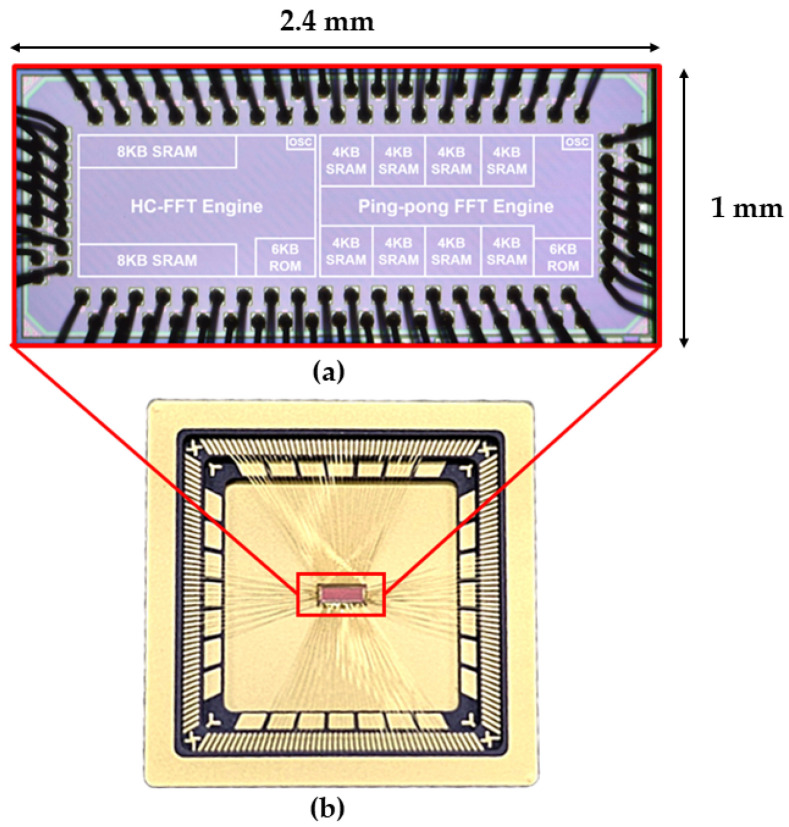
ASIC-based FFT processor: (**a**) annotated die micrograph showing the internal architecture of the 65 nm FFT ASIC (2.4 × 1 mm) and (**b**) the FFT ASIC die.

In parallel, this study investigates an ASIC-based FFT processor to further reduce signal processing power consumption. The FFT ASIC was fabricated using a 65 nm process and has a die size of 2.4 × 1 mm. It implements the same FFT algorithm as the digital board. Functional testing on a dedicated test board confirmed the successful operation of the FFT ASIC die in a SSCM configuration; this result supports the feasibility of the ASIC-based FFT stage for the SSCM signal processing chain. Figure 9a shows the internal architecture of the FFT ASIC die, and Figure 9b shows the wire-bonded die mounted in its package. Ongoing work focuses on system-level integration and verification within the full SSCM electronics stack.

The SCE unit distributes regulated power to each board using an LVPS with flight heritage. A unified 1U CubeSat board size supports integration on multiple platforms, while the digital board applies the Radix-2^2^ FFT algorithm to improve computational efficiency in the on-board signal processing chain. The ongoing study targets additional power reduction by migrating the FFT stage to an ASIC implementation.

## 3. Test Method

The SSCM aims to observe electromagnetic waves in the near-Earth region and the lunar environment. It covers 10 Hz to 20 kHz and targets an NEMI below 1 pT/√Hz at 1 kHz. The SSCM adopts a rolled-sheet core and a bobbin-to-bobbin PCB interconnect, which are less common in flight-qualified SCM implementations. The mission applicability of these designs must be validated under space-relevant conditions.

The SSCM tests comprised performance tests, including frequency response and NEMI tests, and environmental tests, consisting of thermal cycling and vibration tests.

### 3.1. Performance Test

The frequency response test characterized the SSCM measurement bandwidth and transfer function. We computed the transfer function as the ratio of the sensor output (V_out_) to the applied magnetic field (B_in_), as defined in Equation (1).

H(f) = V_out_(f)/B_in_(f) (1)

**Figure 10:**
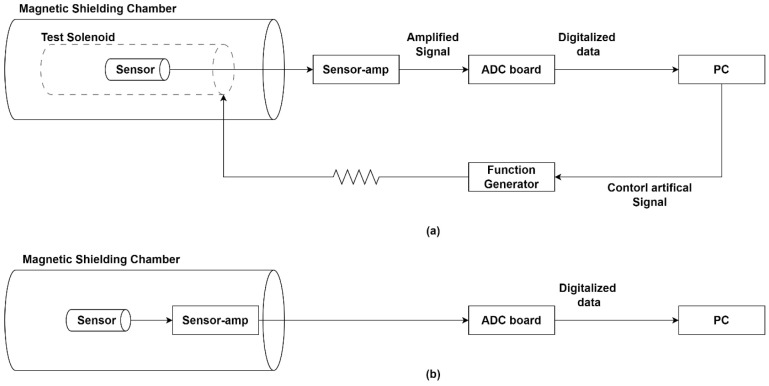
Performance test configuration: (**a**) frequency response test; (**b**) NEMI test.

The frequency test was performed inside a triple-layer mu-metal chamber providing approximately 99% magnetic shielding effectiveness at 1 kHz, which significantly reduces external magnetic interference and enables stable measurement of the sensor response under controlled conditions. The solenoid had a diameter of 80 mm, a length of 1500 mm, and 360 turns of AWG 18 wire. A function generator supplied a sinusoidal voltage to ensure stable excitation. The signal was applied through a 360 kΩ series resistor, which limited the current flowing through the solenoid and allowed the generation of a controlled low-amplitude magnetic field in the nanotesla range, comparable to typical wave amplitudes observed in space environments. The PC linearly swept the sine wave frequency from 10 Hz to 20 kHz with a step of 50 Hz. A commercial PNI sensor placed at the solenoid center provided a coil constant of 301 nT/mA, which was used to calibrate the magnetic field at the solenoid. The frequency response setup is shown in Figure 10a.

Ground tests limit absolute noise verification at the fT/√Hz level, while residual magnetic field variations, laboratory electromagnetic interference, and practical shielding constraints set the achievable noise floor. In this study, NEMI serves as an index for stability and relative noise behavior across the entire measurement frequency range under a fixed configuration. This approach supports the verification of the minimum requirement and the assessment of consistency across the full frequency range. NEMI is computed by dividing the measured output voltage noise spectrum V_n_ by the system transfer function H, as defined in Equation (2).

NEMI(f) = V_n_(f)/|H(f)| (2)

Figure 10b shows the NEMI measurement setup. The sensor and sensor amplifier were placed inside a magnetic shielding chamber to suppress external interference. Measurements were collected during nighttime to further reduce ambient noise. The output voltage noise was continuously acquired using a Lab Jack T7 data acquisition system operated in streaming mode. The acquired data were segmented into 1 s blocks and processed using FFT-based spectral analysis to obtain the voltage noise power spectral density (PSD).

A stable noise estimate was obtained by repeating the measurement under identical conditions and averaging the resulting spectra. For selected frequency bins, PSD values were accumulated over 60 acquisitions and averaged to derive the final voltage noise PSD, which reduced the influence of random fluctuations and transient disturbances. Residual interference may still remain due to ground test conditions despite shielding and harness mitigation. Therefore, NEMI was evaluated across the full frequency range rather than at isolated frequencies, enabling a consistent and reproducible evaluation of the system noise performance under ground test limitations and a reliable basis for NEMI assessment.

**Figure 11:**
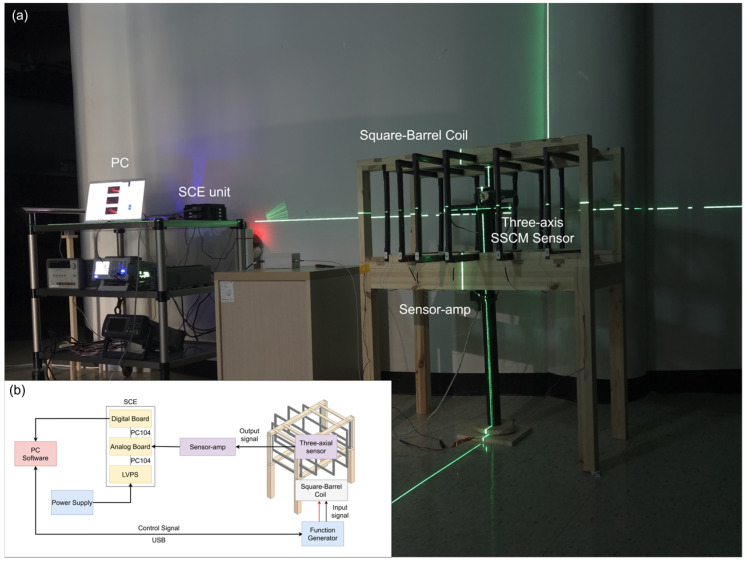
(**a**) Experimental setup used for the SSCM characterization. The three-axis SSCM sensor is positioned at the center of the square barrel coil to measure the controlled magnetic field generated by the coil. The sensor output is processed by the sensor amplifier and the SCE unit and recorded on a PC. (**b**) System configuration diagram of the measurement setup.

Accurate vector magnetic field measurements require stable inter-axis orthogonality and low crosstalk. Although the SSCM was structurally designed to achieve an orthogonal configuration, small angular deviations can arise during assembly and integration. Therefore, additional three-axis evaluations were conducted to quantify the effective inter-axis alignment and to assess measurable crosstalk among the axes. Figure 11 illustrates the experimental configuration for the three-axis characterization. The three-axis SSCM sensor is positioned at the center of the square barrel coil to measure the controlled magnetic field generated by the coil. The sensor output is processed by the sensor amplifier and the SCE unit and recorded on a PC. The inset shows the system configuration of the measurement setup. The SSCM was exposed to a controlled magnetic field environment under sinusoidal excitation at 1 kHz with a magnetic field amplitude of 5.2 nT, and the relative amplitudes and phase responses of the three axes were analyzed to enable the quantitative estimation of orthogonality and the identification of potential crosstalk effects at the system level. In addition, the three-axis characterization was conducted using controlled rotation of the sensor within the uniform magnetic field, and the measured signals were averaged over fixed time intervals to reduce environmental fluctuations and improve statistical reliability. These procedures ensure that the reported orthogonality and crosstalk results reflect stable system-level behavior while avoiding the dependence on transient noise conditions. The detailed calibration procedure and coil configuration will be reported separately.

### 3.2. Environmental Test

Environmental tests were performed to evaluate mechanical and functional stability under space-relevant conditions. The purpose of these tests was to verify that the SSCM maintains structural stability and a stable electrical performance under launch and on-orbit thermal environments.

**Figure 12:**
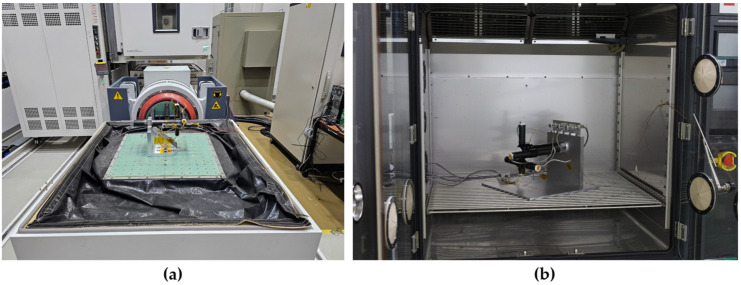
Environmental test configurations: (**a**) vibration test (X: parallel to the boom axis and vibration direction, Y: perpendicular to them); (**b**) thermal cycling test.

A vibration test was used to assess mechanical stability under launch environments. The test sequence followed the vibration requirements of the Korea Pathfinder Lunar Orbiter (Danuri, which launched on a Falcon 9 vehicle), comprising sine sweep–random vibration–sine sweep, and thereby represents a realistic launch vibration environment for space missions. In this study, the vibration test was conducted in two configurations: the X-direction, defined as parallel to the boom axis and the vibration direction, and the Y-direction, defined as perpendicular to the boom axis and the vibration direction. Each configuration was tested independently, and acceleration sensors were attached to each axis during the test to monitor the vibration response. A digital multimeter tracked the coil resistance throughout the test to monitor coil breakage. In addition to resistance monitoring, the system-level frequency response was evaluated after completing both the vibration test and the thermal cycling test to verify overall signal chain performance. The sine sweeps and random vibration conditions are summarized in Table 2 and Table 3, respectively. The environmental test configurations are shown in Figure 12.

**Figure 13:**
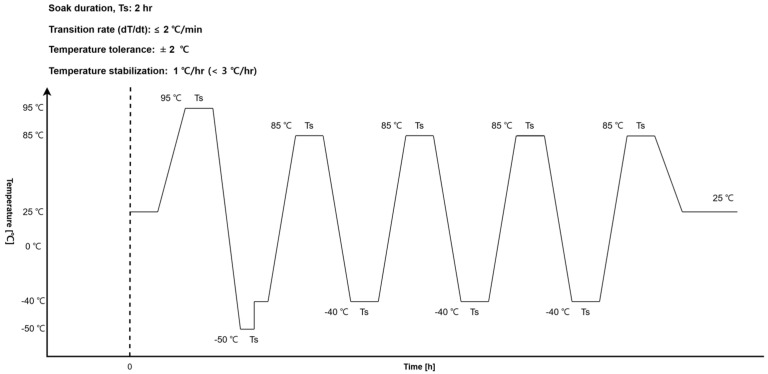
SSCM thermal cycling test temperature profile.

A thermal cycling test was used to verify sensor stability under repeated temperature variation. This test evaluated the structural stability of the core–winding structure under temperature-induced stress. The procedure followed NASA GEVS. The qualification test cycled the temperature from −50 °C to 95 °C for one cycle. The flight acceptance temperature test then cycled the temperature from −40 °C to 85 °C for 3.5 cycles. The soak duration was maintained for 2 h at each temperature hold stage. A temperature sensor attached to the coil and the sensor PCB measured the temperature at 5 s intervals during the test, while coil resistance was recorded at 1 min intervals throughout the test. The thermal profile is shown in Figure 13.

Acceptance criteria were defined as follows: (1) no electrical discontinuity in the coil, (2) no structural damage of the sensor assembly, and (3) no significant deviation in the frequency response characteristics after environmental exposure. These criteria are consistent with standard verification approaches for spaceborne magnetic sensors under ground-based qualification conditions.

## 4. Test Results

### 4.1. Performance Test Results

**Figure 14:**
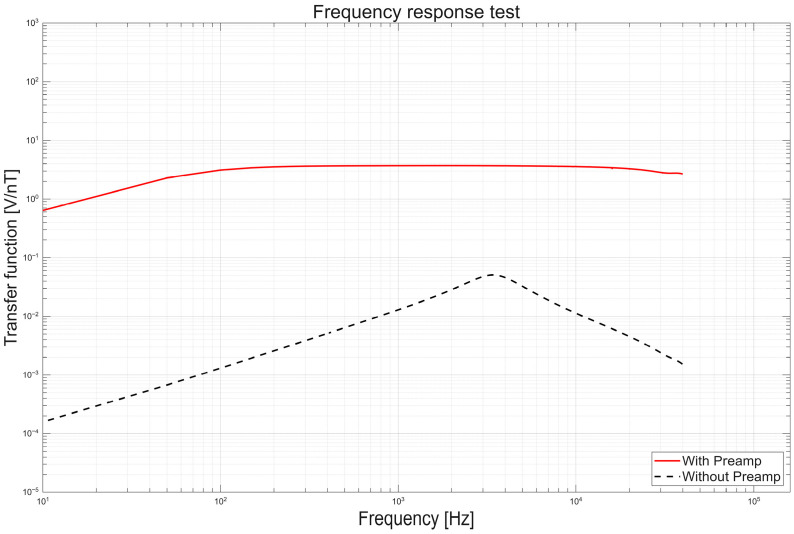
Frequency response test results.

The frequency response test showed the stable operation of the X-axis SSCM sensor of the three-axis SSCM across 10 Hz to 20 kHz. The sensor exhibited a resonance near 3.5 kHz without the sensor-amp. At this resonance, the transfer function increased by about 38 times relative to the value at 100 Hz. With the sensor-amp, the transfer function increase at the resonance frequency was limited to about 1.2 times and the response remained flat above 100 Hz. The sensor-amp provided about 80 dB of gain while suppressing the excessive resonance response by about 30 dB. This result indicates that the SSCM operates stably over a frequency range covering wave phenomena reported in near-Earth and lunar environments. Figure 14 shows the frequency response test results.

**Figure 15:**
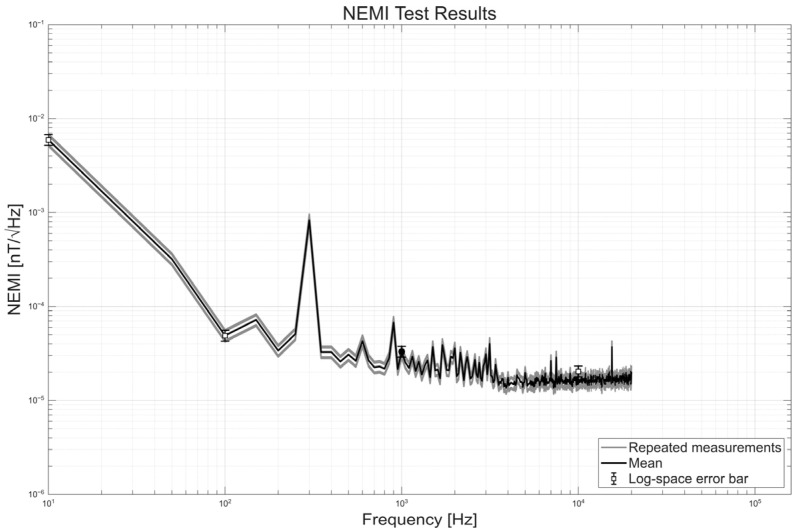
NEMI test results of the SSCM from 10 Hz to 20 kHz. Thin lines show repeated measurements; the solid line is the mean; and error bars indicate log-space variability. At 1 kHz, the mean NEMI is 32.99 fT/√Hz.

The NEMI was computed by dividing the square root of the PSD by the transfer function magnitude obtained from the frequency response test. At 1 kHz, the SSCM achieved an NEMI of 33 fT/√Hz, which met the requirements. To address the limitation that absolute fT-level noise cannot be strictly verified in a ground environment, uncertainty was quantified through repeated measurements over the full frequency range. Figure 15 shows individual NEMI spectra together with their mean and log-space error bars. At 1 kHz, the mean NEMI is 32.99 fT/√Hz, which represents the system-level performance of the complete measurement chain under the present ground test configuration, with a variation range of 28.91–37.65 fT/√Hz (≈±15%), reflecting residual environmental noise and system-level fluctuations rather than intrinsic sensor instability. Despite this variation, the consistent spectral shape across repeated measurements indicates stable operation over 10 Hz to 20 kHz and supports the feasibility of observing wave phenomena at amplitudes of several picotesla. A noise peak near 300 Hz was observed, likely caused by external interference in the ground test environment such as the power system and instrumentation. Further tests with improved power isolation and additional shielding are planned to investigate its origin.

**Figure 16:**
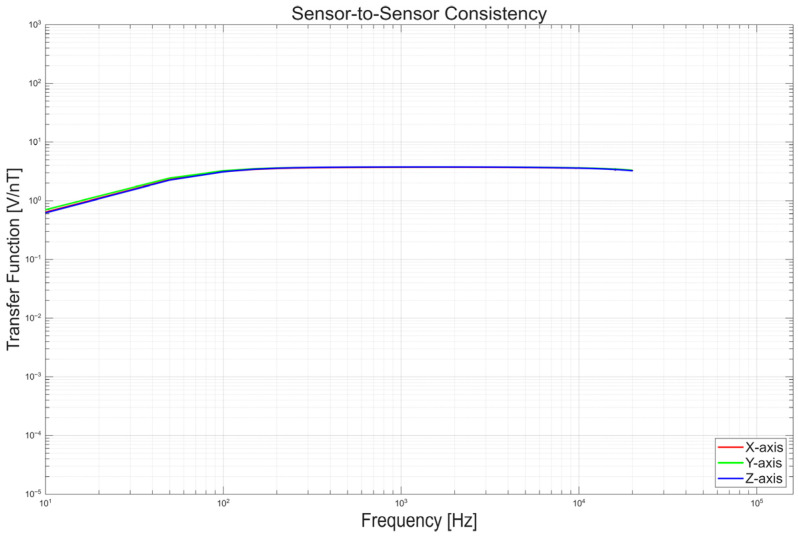
Frequency response comparison of the three sensors.

Before assessing the integrated three-axis characteristics, the consistency of the individual sensors was verified. Figure 16 demonstrates that the three sensors exhibit highly consistent frequency responses under identical conditions (correlation coefficients of 0.9964–0.9985), ensuring that subsequent three-axis results reflect structural and assembly effects rather than intrinsic sensor variation.

**Figure 17:**
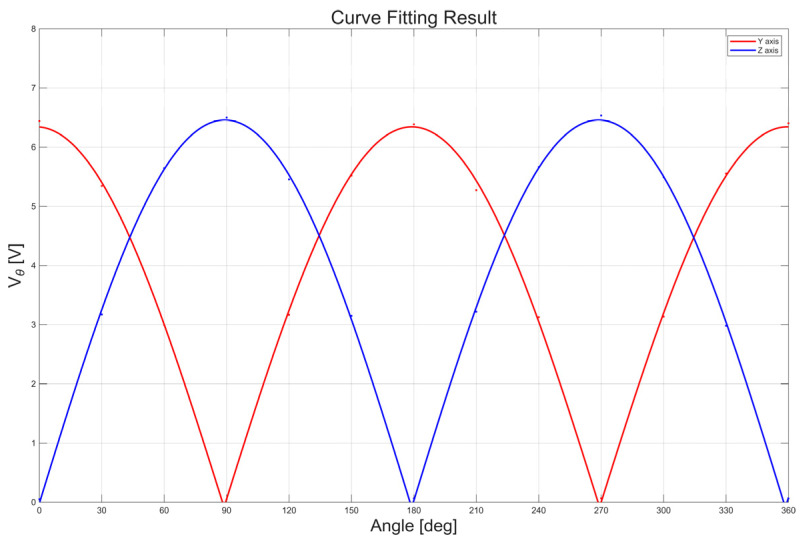
Orthogonality evaluation results based on rotation measurements and curve fitting. The measured sensor outputs as a function of rotation angle are shown together with the fitted curves used to derive the inter-axis phase difference.

Based on this consistency, the integrated three-axis performance was assessed under controlled excitation at 1 kHz with a magnetic field amplitude of 5.2 nT. The crosstalk characteristics were first evaluated by comparing sensor outputs with and without the presence of the magnetic core under identical conditions. Table 4 summarizes the averaged sensor outputs for each condition. The observed variation in the affected axis was approximately 0.002 V, while the environmental noise level was approximately 0.03 V. Since the crosstalk-induced variation is more than one order of magnitude smaller than the background noise, the mutual interference between adjacent sensors can be considered negligible for system-level analysis under the present configuration. The orthogonality between sensor axes was evaluated using rotation-based measurements and phase analysis. Figure 17 shows the measured responses as a function of rotation angle together with the fitted curves. The phase difference between the sensor responses corresponds to an inter-axis angle of approximately 89.17°, indicating that the assembled three-axis structure maintains orthogonality within 1° of the ideal 90° configuration.

These results demonstrate that the three-axis SSCM provides reliable vector magnetic field measurements. The agreement between the measured responses and the expected orthogonal behavior confirms that fabrication and assembly tolerances do not significantly degrade system-level performance.

### 4.2. Environmental Test Results

The environmental tests showed no mechanical and electrical anomalies in the SSCM sensor assembly. The first-mode natural frequency identified during the vibration test changed by less than 5% after the test. Table 5 presents the first-mode natural frequency results of the sine sweep tests prior to and following the random vibration test. No structural damage or electrical discontinuity was observed during the environmental tests. The system-level frequency response measured after completion of the environmental tests confirmed stable operation, with variations within approximately 5% over the measurement frequency range from 1 Hz to 20 kHz.

### 4.3. Compared with Spaceborne Search Coil Magnetometers

Table 6 compares the SSCM with representative spaceborne search coil magnetometers used in previous missions. Previous SCM instruments were typically developed for large or medium-class spacecraft platforms where sensor mass and system resources were relatively less constrained and therefore prioritized high sensitivity and extended frequency coverage for dedicated plasma wave investigations. The table summarizes the key design and performance parameters of representative SCM instruments, including observation frequency range, noise-equivalent magnetic induction (NEMI), core length, sensor mass, and electronic power, providing context for the SSCM design presented in this work.

Sensor mass values reported in the literature are not always defined using the same criteria. Some missions report the mass of a single sensor element, whereas others report the mass of the three-axis sensor assembly including the supporting structure. The SSCM mass corresponds to the mass of a single-axis sensor. Similarly, the power value reported for the SSCM represents the total system-level electronic power consumption, including the sensor amplifier, signal digitization, and on-board signal processing. In contrast, the power values reported for several previous missions typically correspond only to the preamplifier stage and do not include the full signal processing chain. Therefore, direct comparison of power consumption should be interpreted with caution, as the SSCM measurement represents an integrated instrument-level configuration rather than a standalone preamplifier stage.

The SSCM in this study focuses on achieving a comparable observation capability while reducing sensor mass and enabling integration with resource-constrained platforms. The SSCM operates over a frequency range of 10 Hz to 20 kHz, which is comparable to the observation bands adopted in several previous spaceborne SCM missions designed for near-Earth plasma wave observations. The measured NEMI of 0.033 pT/√Hz at 1 kHz is also within the range reported for many spaceborne SCM instruments under ground-based validation conditions.

Compared to previous systems, the SSCM achieves a higher core-length-to-mass ratio with a 0.23 m core and 0.12 kg sensor while the reported 3.92 W represents a fully integrated acquisition and processing chain rather than a preamplifier-only figure. A key design objective of the SSCM was achieving efficient magnetic flux coupling while reducing sensor mass. This combination enables efficient magnetic flux coupling without significantly increasing payload mass. The mass reduction is primarily achieved through the rolling-sheet core configuration which reduces core weight while preserving the required magnetic response characteristics.

In addition to the favorable mass-to-performance characteristics, the SSCM architecture includes system-level integration. Unlike several previous SCM implementations where signal digitization and processing are performed by separate spacecraft subsystems, the SSCM integrates the sensor amplifier, ADC, and digital signal processing electronics within a unified architecture. This configuration enables the SSCM to operate as an integrated standalone measurement system that can be directly interfaced with platform avionics while minimizing additional payload resources.

Overall, these characteristics indicate that the SSCM architecture provides a practical design approach for implementing broadband magnetic field measurements in missions where payload mass and system resources are limited, such as small satellite or CubeSat-class platforms.

## 5. Conclusions

The SSCM was developed to observe electromagnetic waves in space while reducing sensor mass and electronic power consumption for resource-constrained space missions. The system maintains a stable frequency response over 10 Hz to 20 kHz and achieves a system-level NEMI of 33 fT/√Hz at 1 kHz. Environmental tests confirmed that the mass-reduced sensor structure maintains mechanical safety and functional stability under space-relevant conditions.

Mass reduction was primarily achieved through adoption of a rolling-sheet core architecture. The rolling-sheet core enables up to an approximately 75% reduction in core mass relative to conventional rod-type cores while preserving the required measurement bandwidth. Power reduction was realized through the ASIC-based sensor amplifier, which reduced total system power consumption from 4.48 W to 3.92 W. Further optimization of the electronics chain is being investigated through the development and integration of an ASIC-based FFT processor to reduce signal processing power consumption at the system level.

This study demonstrates a system-level design methodology for compact spaceborne search coil magnetometers, in which sensor structure, amplification, and signal-processing components are co-designed to balance sensitivity, bandwidth, sensor mass, and power under platform constraints. The SSCM therefore focuses on achieving a balanced trade-off between sensitivity, bandwidth, sensor mass, and power consumption for platform-constrained missions while maintaining a practical measurement capability across the target observation band. The proposed low-mass and power-efficient SSCM design offers a practical implementation approach for broadband magnetic field measurements on spacecraft operating under strict resource constraints. In this context, the SSCM architecture presented in this study can serve as one possible methodological approach for implementing search coil magnetometers on future spacecraft platforms, including lunar missions and other space exploration missions of various scales.

## Figures and Tables

**Table 1:** SSCM requirements.

Item	Requirements	
Performance	Frequency Range	From 10 Hz to 20 kHz
	NEMI	<1 pT/√Hz at 1 kHz
System	Total Mass	<9 kg
	Input Voltage	Input: 28 V
	Interface	RS-422
	Operating Temperature	MAG unit: −55–+70 °C SCE unit: −20–+50 °C

**Table 2:** Sine sweep test requirements.

Item	Contents
Type	Harmonic
Amplitude	0.4 g (peak acceleration applied during sine excitation)
Frequency range	5–2000 [Hz]
Sweep rate	0.6 [decade/min] (equivalent to 2 oct/min)

**Table 3:** Random vibration test requirements.

Item		Contests
Profile	Frequency range [Hz]	Amplitude [g^2^/Hz]
	10–60	+9 dB/oct
	60–400	0.22
	400–2000	−6 dB/oct
RMS Acceleration		12.3 [g]
Time duration		2 min per direction

**Table 4:** Crosstalk evaluation results based on averaged sensor outputs with and without the magnetic core under excitation conditions.

Case	Y Axis	Z Axis
Case 1 (With core)	4.4526	4.4533
Case 2 (Without core)	4.4532	0.2699
Case 3 (With core)	4.4511	4.4541

**Table 5:** First-mode natural frequency before and after the random vibration test for each measurement direction, showing variations within 5%.

	Pre-Random	Post-Random	Variation
Axial	138	141	2.17
Perpendicular	139	143	2.87
Radial	603	629	4.31

**Table 6:** Comparison with representative spaceborne search coil magnetometers used in previous missions.

Mission	Frequency Range	NEMI [pT/√Hz] (Representative)	Core Length [m]	Mass [kg]	Power [W]
DEMETER [24]	10 Hz–20 kHz	0.004 at 6 kHz	0.17	0.1 (single sensor)	0.08 (preamplifier)
THEMIS [14]	0.1 Hz–4 kHz	0.02 at 1 kHz	0.17	0.568 (including sensor structure)	0.075 (preamplifier)
Van Allen Probes [11]	10 Hz–12 kHz	0.005 at 3 kHz	0.40	N/A	N/A
MMS [25]	0.1 Hz–6 kHz	0.025 at 1 kHz	0.10	0.239 (including sensor structure)	0.13 (preamplifier)
Arase [26]	A few Hz–20 kHz	0.02 at 2 kHz	0.20	0.09 (single sensor)	0.43 (electronics)
CANVAS [27]	0.3–40 kHz	0.07	0.10	N/A	N/A
SSCM	10 Hz–20 kHz	0.033 at 1 kHz	0.23	0.12 (single sensor)	3.92 (system)

N/A: Not available.

## Data Availability

The datasets supporting the findings of this study are available in the Zenodo repository at https://doi.org/10.5281/zenodo.19480622 (accessed on 9 April 2026).

## References

[1] Roberts D., Shelhamer M., Wong A. (2008). A new “wireless” search-coil system. Proceedings of the 2008 Symposium on Eye Tracking Research & Applications, Savannah, GA, USA, 26–28 March 2008.

[2] Poliakov S.V., Reznikov B.I., Shchennikov A.V., Kopytenko E.A., Samsonov B.V. (2017). The range of induction-coil magnetic field sensors for geophysical explorations. Seism. Instrum..

[3] Ertan H.B., Keysan O. (2009). External search coil as a means of measuring rotor speed of an induction motor. Proceedings of the 2009 8th International Symposium on Advanced Electromechanical Motion Systems & Electric Drives Joint Symposium, Lille, France, 1–3 July 2009.

[4] Thorne R.M. (2010). Radiation belt dynamics: The importance of wave-particle interactions. Geophys. Res. Lett..

[5] Kennel C.F., Petschek H.E. (1966). Limit on stably trapped particle fluxes. J. Geophys. Res..

[6] Tsurutani B.T., Falkowski B.J., Pickett J.S., Verkhoglyadova O.P., Santolik O., Lakhina G.S. (2014). Extremely intense ELF magnetosonic waves: A survey of polar observations. J. Geophys. Res. Space Phys..

[7] Santolík O., Gurnett D.A., Pickett J.S., Parrot M., Cornilleau-Wehrlin N. (2003). Spatio-temporal structure of storm-time chorus. J. Geophys. Res. Space Phys..

[8] Thorne R.M., Smith E.J., Burton R.K., Holzer R.E. (1973). Plasmaspheric hiss. J. Geophys. Res..

[9] Abel B., Thorne R.M. (1998). Electron scattering loss in Earth’s inner magnetosphere: 2. Sensitivity to model parameters. J. Geophys. Res. Space Phys..

[10] Horne R.B., Thorne R.M., Glauert S.A., Meredith N.P., Pokhotelov D., Santolík O. (2007). Electron acceleration in the Van Allen radiation belts by fast magnetosonic waves. Geophys. Res. Lett..

[11] Kletzing C.A., Kurth W.S., Acuna M., Macdowall R.J., Torbert R.B., Averkamp T., Bodet D., Bounds S.R., Chutter M., Connerney J. (2013). The Electric and Magnetic Field Instrument Suite and Integrated Science (EMFISIS) on RBSP. Space Sci. Rev..

[12] Lee H., Jin H., Jeong B., Lee S., Lee S., Baek S.-M., Shin J., Lee J.-K., Park H., Kim K.-H. (2021). KMAG: KPLO Magnetometer Payload. Astron. Soc. Pac..

[13] Auster H.U., Glassmeier K.H., Magnes W., Aydogar O., Baumjohann W., Constantinescu D., Fischer D., Fornacon K.H., Georgescu E., Harvey P. (2008). The THEMIS Fluxgate Magnetometer. Space Sci. Rev..

[14] Roux A., Contel L.O., Coillot C., Bouabdellah A., La Porte D.B., Alison D., Ruocco S., Vassal M.C. (2008). The Search Coil Magnetometer for THEMIS. Space Sci. Rev..

[15] Ripka P. (1992). Review of fluxgate sensors. Sens. Actuators A Phys..

[16] Korepanov V., Marusenkov A. (2012). Flux-Gate Magnetometers Design Peculiarities. Surv. Geophys..

[17] Hospodarsky G.B. (2016). Spaced-based search coil magnetometers. J. Geophys. Res. Space Phys..

[18] Brunke H.-P., Widmer-Schnidrig R., Korte M. (2017). Merging fluxgate and induction coil data to produce low-noise geomagnetic observatory data meeting the INTERMAGNET definitive 1 s data standard. Geosci. Instrum. Methods Data Syst..

[19] Shin J., Kim K.-H., Jin H., Kim H., Kwon J.-W., Lee S., Lee J.-K., Lee S., Jee G., Lessard M.R. (2016). Development of Ground-Based Search-Coil Magnetometer for Near-Earth Space Research. J. Magn..

[20] Kang H., Jin H., Jang Y., Lee S., Park H., Kim J., Jo W. (2024). Performance Test of Search Coil Sensors with Different Core Types. J. Astron. Space Sci..

[21] Kim T., Ryu G., Lee J., Cho M.-K., Fleetwood D.M., Cressler J.D., Song I. (2024). Simple Modeling and Analysis of Total Ionizing Dose Effects on Radio-Frequency Low-Noise Amplifiers. Electronics.

[22] Zhou L., An J. (2013). Design of a Payload Data Handling System for Satellites.

[23] Hussain M.Z., Parvin K.N. (2019). Low power and high performance FFT with different radices. Int. J. Reconfig. Embed. Syst..

[24] Séran H.C., Fergeau P. (2005). An optimized low-frequency three-axis search coil magnetometer for space research. Rev. Sci. Instrum..

[25] Contel L.O., Leroy P., Roux A., Coillot C., Alison D., Bouabdellah A., Mirioni L., Meslier L., Galic A., Vassal M.C. (2016). The Search-Coil Magnetometer for MMS. Space Sci. Rev..

[26] Ozaki M., Yagitani S., Kasahara Y., Kojima H., Kasaba Y., Kumamoto A., Tsuchiya F., Matsuda S., Matsuoka A., Sasaki T. (2018). Magnetic Search Coil (MSC) of Plasma Wave Experiment (PWE) aboard the Arase (ERG) satellite. Earth Planets Space.

[27] Marshall R.A., Reid R.A., Cannon J.M., Wankmueller S., Vankawala P., Palo S., Malaspina D.M., de Wit T.D., Jannet G. The CANVAS Mission: Quantifying the Very-Low-Frequency Radio Energy Input from the Ground into the Earth’s Magnetosphere. Proceedings of the 36th Annual Small Satellite Conference.

